# An Extraction Tool for Venous Thromboembolism Symptom Identification in Primary Care Notes to Facilitate Electronic Clinical Quality Measure Reporting: Algorithm Development and Validation Study

**DOI:** 10.2196/63720

**Published:** 2025-08-26

**Authors:** John Novoa-Laurentiev, Mica Bowen, Avery Pullman, Wenyu Song, Ania Syrowatka, Jin Chen, Michael Sainlaire, Frank Chang, Krissy Gray, Purushottam Panta, Luwei Liu, Khalid Nawab, Shadi Hijjawi, Richard Schreiber, Li Zhou, Patricia C Dykes

**Affiliations:** 1 Department of Medicine, Brigham & Women's Hospital, 75 Francis Street, Boston, MA, 02115, United States, 1 8572824088; 2 Department of Medicine, Harvard Medical School, Boston, MA, United States; 3 Department of Medicine, University of Alabama at Birmingham, Birmingham, AL, United States; 4 Department of Biomedical Informatics, University of Kentucky, Lexington, KY, United States; 5 Department of Information Services, Penn State Health, Hershey, PA, United States; 6 Department of Medicine, Penn State College of Medicine, Hershey, PA, United States; 7 Department of Biomedical Informatics and Data Science, Johns Hopkins School of Medicine, Baltimore, MD, United States

**Keywords:** natural language processing, venous thromboembolism, electronic clinical quality measure, timely diagnosis, primary care, NLP, thromboembolism, clinical quality, algorithm development, algorithm validation, VTE, diagnosis, primary care, extraction tool, tool, clinical note, extraction, AI, artificial intelligence

## Abstract

**Background:**

Diagnosis of venous thromboembolism (VTE) is often delayed, and facilitating earlier diagnosis may improve associated morbidity and mortality. Clinical notes contain information not found elsewhere in the medical record that could facilitate timely VTE diagnosis and accurate quality measurement. However, extracting relevant information from unstructured clinical notes is complex. Today, there are relatively few electronic clinical quality measures (eCQMs) in our national payment program and none that use natural language processing (NLP) techniques for data extraction. NLP holds great promise for making quality measurement more accurate and more efficient. Given the potential of NLP-based applications to facilitate more accurate VTE detection, primary care is one clinical setting in urgent need of this type of tool.

**Objective:**

This study aimed to develop a tool that extracts VTE symptoms from clinical notes for use within an eCQM to quantify the rate of delayed diagnosis of VTE in primary care settings.

**Methods:**

We iteratively developed an NLP-based data extraction tool, venous thromboembolism symptom extractor (VTExt), on an internal dataset using a rule-based approach to extract VTE symptoms from primary care clinical note text. The VTE symptoms lexicon was derived and optimized with physician guidance and externally validated using datasets from 2 independent health care organizations. We performed 26 rounds of performance evaluation of notes sampled from the case cohort (17,585 patient progress note sentences from 279 patient notes), and 5 rounds of evaluation of the control cohort (2838 patient progress note sentences from 50 patient notes). VTExt’s performance was evaluated using evaluation metrics, including area under the curve, positive predictive value, negative predictive value, sensitivity, and specificity.

**Results:**

VTExt achieved near-perfect performance in extracting VTE symptoms from primary care notes sampled from records of patients diagnosed with or without VTE. In external validation, VTExt achieved promising performance in 2 additional geographically distant organizations using different electronic health record systems. When compared against a deep learning model and 4 machine learning models, VTExt exhibited similar or even improved performance across all metrics.

**Conclusions:**

This study demonstrates a data-driven NLP-based approach to clinical note information extraction that can be generalized to different electronic health record systems across different institutions. Due to the robust performance of this tool, VTExt is the first NLP application to be used in a nationally endorsed eCQM.

## Introduction

Venous thromboembolism (VTE) is an often undetected condition that includes both deep vein thrombosis (clots in the deep veins of the body [1]), and pulmonary embolism (PE; clot breaking free and entering the pulmonary arteries [1,2]). VTE is associated with increased morbidity and mortality [3] with a 1-year VTE case-fatality rate estimated at 23% [4] and associated with increased health care costs [5].

The incidence of VTE in the United States is unknown as there is currently no national VTE surveillance system in place [1]. Cases are often missed since they are asymptomatic or associated with symptoms similar to those of other chronic conditions, leading to substantial undercounting. In a 2015 literature review, Heit [6] identified the incidence of VTE as ranging from 104 to 183 cases per 100,000 person-years. This rate is based largely on Caucasian populations [4,7-16] and differs by race where African American individuals face higher rates of VTE [17-19], and Asian [20], Asian American [21,22], and Native American individuals [23] see a lower VTE incidence. Higher levels of education, income, and employment status have also been shown to be associated with decreased risk of VTE [24]. Risk factors for VTE include a history of VTE [25] (rates of recurrent VTE range from 20%‐36% within 10 years of the initial VTE event [26,27]), older age [1], recent immobility or surgery, cancer, smoking, thrombophilia [28], and obesity [6].

Delayed diagnosis of VTE is common due to its nonspecific symptoms [29]. VTE can also be difficult to identify in the electronic health record (EHR) due to variability in how VTE is documented and coded [30]. Due to these challenges and the lack of national surveillance, the incidence of VTE is likely underestimated [31,32]. Tools to facilitate measurement and earlier diagnoses of VTE may help in better understanding VTE risk factors, reduce associated morbidity and mortality [33,34], and improve patient safety.

The widespread adoption of interoperable EHR systems after the 2009 Health Information Technology for Economic and Clinical Health Act [35,36] has led to a significant increase in unstructured text data, such as radiology reports, progress notes, and discharge summaries [37]. These unstructured data are estimated to constitute over 80% of health and biomedical information [37]. Free-text clinical notes in EHRs hold valuable insights for population-level quality improvement, but efficient strategies leveraging AI, machine learning, and natural language processing (NLP) are essential to harness this potential.

NLP is useful for analyzing unstructured EHR data in areas like radiology [38], oncology [39,40], endocrinology [41], substance misuse [42], PE identification [43], and postoperative VTE [44]. By extracting information from text, NLP creates structured data, reducing manual review and enabling large-scale automated processing [45]. High-throughput phenotyping algorithms using NLP-derived and structured data show promise for developing standardizing labeling [46] particularly for managing complex diseases in large-scale patient populations. NLP can also uncover critical information overlooked using structured variables [47,48]. While large language models (LLMs) are popular for NLP tasks, they are often more resource-intensive and costly than traditional machine learning or rule-based methods [49]. Though machine learning methods tend to have improved performance, a rule-based approach has advantages, such as traceability of results and speed of development [50].

NLP tools can detect VTE events, but more sensitive tools are needed to identify VTE events specifically in primary care EHR progress notes [44,51]. The objective of this study was to develop a simple, accessible NLP tool for identifying VTE symptoms in primary care EHRs, suitable for both high- and low-resource settings and aligned with the national quality payment program. The tool was tested on external datasets to evaluate its performance compared with deep learning and machine learning models. This main aim is to use narrative EHR data for clinical quality reporting to identify missed or delayed diagnoses of VTE after a primary care visit. A delayed diagnosis is defined as one that occurs >24 hours after the primary care visit when the VTE symptoms were documented.

## Methods

### Data Sources, Cohort Development, and Feature Selection Strategy

The study was conducted at Mass General Brigham (MGB), an integrated health care delivery system in Greater Boston, Massachusetts, using data from the MGB Enterprise Data Warehouse (EDW), an MGB central clinical data warehouse.

We used 2 internal datasets to develop and evaluate our NLP application for symptom extraction, and 2 independent external datasets to test how well it works in other settings. The first internal dataset, the case cohort, was used for development and evaluation. Inclusion criteria for this cohort are described below. The second internal dataset, the control cohort, included patients who did not meet case cohort inclusion criteria and was used for further evaluation. The external validation datasets came from 2 university health systems: the University of Kentucky and Penn State Health. These datasets were used to test if our symptom extractor works well with notes from different EHR vendors and health care systems.

We developed a multifactor phenotyping algorithm to identify VTE patients in the MGB cohort [52]. This included patients diagnosed with VTE from 2016 through 2021 who had a primary care visit in the 30 days before the date of diagnosis. We started by using *ICD-10* (*International Statistical Classification of Diseases, Tenth Revision*) codes to identify an initial VTE patient cohort. Then we combined data from imaging records (eg, current procedural terminology [CPT] codes) and anticoagulant orders (RxNorm codes) to further refine the initial cohort and develop the final VTE case cohort. The diagnosis date and time of VTE diagnosis was defined as when the radiologist signed off on the scan report [52,53].

We used a rule-based approach to identify terms from a lexicon derived from a set of VTE signs and symptoms. The lexicon was divided into 3 parts: one with relevant symptoms dependent on the part of the body (eg, swelling), another with the relevant symptom locations (eg, leg), and the last containing location-independent symptoms (eg, cough). Location-dependent symptoms required identification of both the symptom and a relevant location to be considered a symptom match. The lexicon was reviewed and revised over the course of the study in accordance with physician expert guidance.

### Clinician-Guided VTE Lexicon Development and Optimization

We identified VTE-related signs and symptoms by combining a literature review with interviews of physicians with experience in treating VTE patients. Multiple optimization steps were conducted: first, we conducted a comprehensive literature review to create an initial list of signs and symptoms. Then, we held 1-hour semistructured interviews with 5 experienced physicians to provide additional insight into signs and symptoms based on clinical experience. Signs and symptoms were also reviewed by a technical expert panel over the course of development, and their feedback was used to finalize the lexicon. In total, we included 29 distinct symptoms in the lexicon, consisting of 7 location-independent symptoms, 7 location-dependent symptoms, and 4 relevant locations. The final VTE symptom 3-part lexicon can be found in Multimedia Appendix 1. Inclusion criteria *ICD-10*, CPT, and RxNorm codes are provided in Multimedia Appendix 2. The prevalence of each symptom in each dataset is provided in Multimedia Appendix 3.

### Extractor Development and Optimization

The Medical Text Extraction, Reasoning and Mapping System (MTERMS) [54] venous thromboembolism symptom extractor (VTExt) was developed using the Python programming language. We chose a rule-based approach to identify symptoms in order to facilitate transferability of the tool and to ensure transparency of its workings, which can be challenging when using more complex machine learning or LLM-based approaches [55]. Using a rule-based approach also suited the need for VTExt to identify VTE symptoms within specific contexts, for example, at specific body locations.

**Figure 1.**
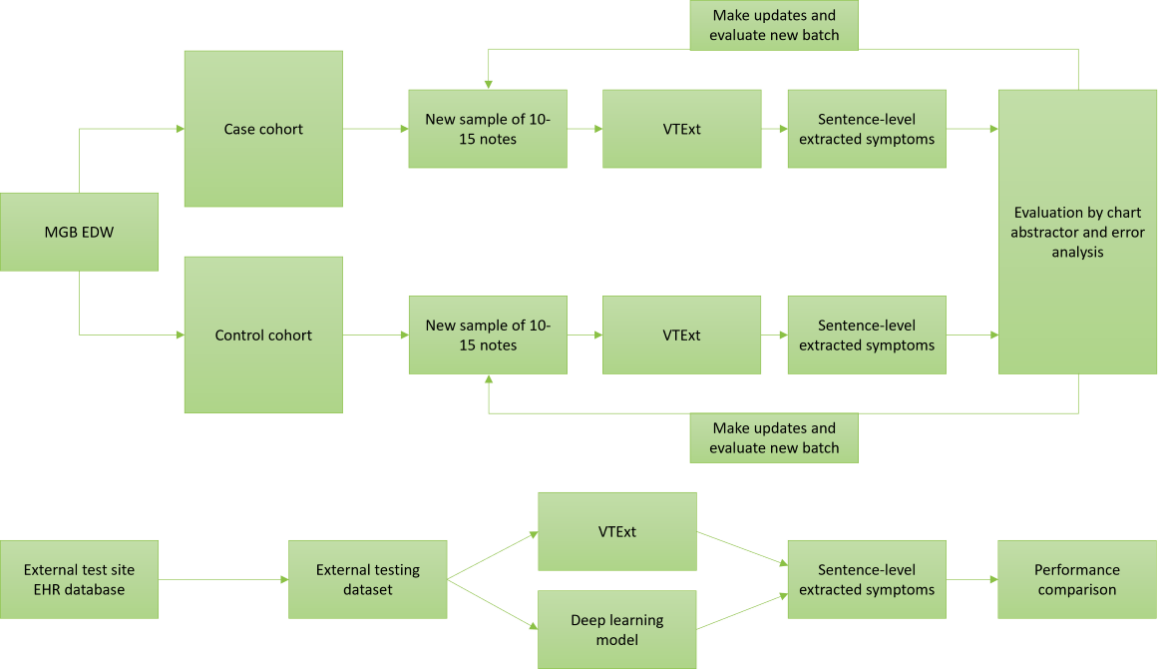
Venous thromboembolism symptom extractor development and evaluation process. EDW; enterprise data warehouse; EHR; electronic health record; MGB; Mass General Brigham; VTExt: venous thromboembolism symptom extractor.

The development cycle used in the creation of VTExt entailed initial analysis of symptom extractor requirements, design and implementation of the extractor, iterative testing on samples of patient notes, and adjusting VTExt based on error analyses. The overall study design and development process is provided in Figure 1.

We first reviewed a small sample of cases from the dataset described above to understand how VTE symptoms appear in primary care progress notes, for example, how providers document VTE symptoms. The initial version of VTExt was then used to extract symptoms from a batch of sampled primary care progress notes. A trained chart abstractor reviewed each sentence analyzed by VTExt and determined whether the structured output was accurate, marking each case as a true positive, true negative, false positive, or false negative. Whenever an error occurred, the reason was identified, and adjustments were made to the extractor to avoid the error in the processing of future batches. We repeated this optimization process of running the extractor on a new sample of 10‐15 notes, reviewing output, and refining the pattern-matching to iteratively improve the performance of the symptom extractor until we achieved a precision (positive predictive value [PPV]) of at least 0.95.

For each round, one progress note from each patient visit was extracted and combined into a single file. Patient notes were split into sentences using the MTERMS NLP system [54]. The symptom extractor then used regular expression-based rules to identify signs and symptoms of VTE in the curated lexicon and wrote output to a structured query language database to allow for integration of extractor output into other pipelines, including mapping symptoms to standardized terminologies. The NLP output table contains one column for each VTE symptom in the lexicon. Each row in the table corresponds to 1 patient note, and a binary output value for each symptom field indicates whether a given symptom was detected in the note by VTExt—if yes, presence was indicated with a value of “1,” and if not, a value of “0.”

To facilitate the clinical implementation of our tool, we developed a streamlined version of VTExt with simplified output for use with the electronic clinical quality measure (eCQM). Instead of producing output values for presence of individual signs and symptoms, this version produced a single “0” or “1” value for each patient note to indicate whether at least 1 VTE symptom was identified. This streamlined version of VTExt was used in the external evaluation of the tool. Pseudocode for the tool can be found on our project GitHub page [56].

### Note Processing and Evaluation

We evaluated the VTExt symptom extractor using both internal and external datasets. For internal evaluation, we used both a case cohort and a control cohort. The case cohort included patients who met our inclusion criteria for incident VTE based on the presence of 3 codes; *ICD-10* VTE codes, CPT imaging codes, and RxNorm anticoagulation codes [53]. The control cohort included patients who did not meet these criteria.

Internal evaluation of the VTExt symptom extractor was an iterative process, illustrated in Figure 1. From all patients who met the case cohort inclusion criteria, we randomly selected batches of 10 to 15 patient visits for each round of testing. We used a similar method to sample control notes to evaluate how well the symptom extractor generalized to patients that did not meet the case cohort inclusion criteria (eg, patients who did not have a VTE diagnosis).

### External Evaluation

We worked with collaborators at both the University of Kentucky and Penn State Health (PSH) to test VTExt on patient notes. These sites used different EHR systems which also differed from MGB and had different textual data structures. In Epic (used at MGB), patient notes exist in tables, which include note-related information including metadata and the note content itself. Veradigm (formerly Allscripts; used by University of Kentucky) and Oracle Cerner (used by PSH) similarly store patient note data in document tables. For free text notes in Veradigm and Oracle Cerner, note contents of many documents are stored in “Character Large Objects” or “Binary Large Objects” fields. Notes in these areas of the database require special querying techniques to extract unstructured text, usually requiring certified analysts. Despite these differences, once note text data are available, the NLP tool functions properly irrespective of the EHR as it is not dependent on the EHR itself.

In addition, each system served a different population: MGB serving mostly urban and metro, University of Kentucky serving more rural, and PSH serving a mixed population of urban, metro, and rural. The diversity of sites included served as a good preliminary test for generalizability of VTExt.

During external evaluation, we compared the performance of the rule-based extractor against a pretrained sequence classification deep learning model derived from Bio+Clinical BERT (bidirectional encoder representations from transformers; using the HuggingFace transformers Python package), a contextualized word representation model based on BioBERT and trained further on Medical Information Mart for Intensive Care (MIMIC) data [57-60]. We also compared performance against 4 classical machine learning models: logistic regression, support vector machine (SVM), and random forest, implemented using the Python Scikit-learn module, and gradient boosting, implemented using the Python XGBoost module [61-63]. MGB data used during the development of VTExt were preprocessed using the Bio+Clinical BERT tokenizer for further training of the deep learning model. For training the four classical models, the MGB data were instead represented as unigrams transformed using term frequency—inverse document frequency (TF-IDF) [64]. For all models, data were divided into training and validation sets for training and tuning of model parameters, respectively. Final parameters for deep learning and machine learning models are provided in Multimedia Appendix 4. Each external site manually labeled a testing set of 500 note sentences for evaluation, 250 containing at least one VTE symptom and 250 with no VTE symptoms.

### Ethical Considerations

This project was reviewed and approved by the Mass General Brigham institutional review board (protocol #2020P003979). In this protocol, a waiver of informed consent and a waiver of HIPAA (Health Insurance Portability and Accountability Act) authorization was requested because this quality improvement research involves no more than minimal risk to the participants and the research could not practicably be carried out without the waiver given the large number of patients who had a VTE diagnosis in a primary care setting. In addition, this research could not practicably be conducted without access to and use of the protected health information. The following procedures were followed to prevent breach in confidentiality: (1) data were accessed only behind MGB firewall using password-protected, secure devices by Collaborative Institutional Training Initiative–certified study staff, and (2) we will destroy all patient identifiers at the end of the study, once analysis and publications are finalized. In accordance with the approved institutional review board protocol, all electronic data were kept in password-protected files on a secure server behind the MGB firewall. Only study personnel were given a unique identifier—no participant identifiers are linked to the data. No compensation was provided for participation.

## Results

We performed 26 rounds of evaluation of VTExt performance on notes sampled from the case cohort. This included 17,585 patient progress note sentences from 279 notes from distinct patients, 171 of which were found to contain 1 or more VTE symptoms. Evaluation of the control cohort included 2838 note sentences from 50 patient notes over the course of five rounds of evaluation, of which 21 notes contained at least 1 relevant symptom.

Performance was evaluated at the sentence level. We measured precision (PPV), recall (sensitivity), specificity, and negative predictive value (NPV; Table 1). Of these metrics, achieving a high precision score proved to be the greatest challenge. Many false positives initially arose due to 3 kinds of errors, shown in Table 2. Some errors were due to word misspellings in the notes (which we refer to as type A errors). For example, misspelling of the word “denies” caused VTExt to miss negation of subsequent VTE symptoms. In other cases, an error occurred because a symptom was identified but was attributed to the incorrect body part (a type B error). Many false positives arose in early stages of evaluation from failure to detect negation or context, as in the Type C error examples in Table 2.

**Table 1.** Venous thromboembolism symptom extractor validation performance on notes of case cohort (patients with venous thromboembolism diagnosis).

Validation round	Patients, n	Precision (positive predictive value)	Recall (sensitivity)	Specificity	Negative predictive value
Round 1	673	0.500	0.863	0.929	0.988
Round 9	692	0.851	0.966	0.984	0.997
Round 17	489	0.750	1.000	0.998	1.000
Round 26	938	1.000	1.000	1.000	1.000

**Table 2.** Examples of common sources of symptom extractor false positive errors.

Error type	Examples
Type A: misspelling	She “deneis” shortness of breath or pleuritic chest pain
Type B: symptom attributed to wrong body part	Worsening R hip “pain” as well as recent development of R “leg,” ankle, and foot erythema
Type C: negation or context	“Resolution” of hypoxia and chest pain. Nitroglycerin 0.4 MG SL tablet place 1 tablet (0.4 mg total) under the tongue every 5 (five) minutes “as needed” for chest pain

For the first example, VTExt captured the symptom hypoxia without identifying the negating phrase “resolution of.” In the second example, though chest pain is mentioned, it appears in the context of a medication to be taken as needed, which we deemed not to be strong enough evidence of the presence of a symptom. Repeated validation allowed us to learn what contexts and negating phrases appeared in clinical text, and this knowledge was used to improve VTExt’s ability to locate them. Through this process, precision improved from 0.5 in the first round of testing to near-perfect in the final round. Near-perfect performance was also achieved for recall, specificity, and NPV in the final round of validation. In addition, we tested the extractor on several random samples of primary care clinical notes of patients in the cohort, that is, those not diagnosed with VTE (Table 3, in batches of 10‐15 notes, with precision ultimately reaching 0.85).

**Table 3.** Venous thromboembolism symptom extractor validation performance on notes of the control cohort (patients with no venous thromboembolism diagnosis).

Validation round	N	Precision (positive predictive value)	Recall (sensitivity)	Specificity	Negative predictive value
Round 1	281	0.533	1.000	0.974	1.000
Round 2	471	0.556	1.000	0.991	1.000
Round 3	613	0.750	1.000	0.998	1.000
Round 4	559	0.806	1.000	0.989	1.000
Round 5	912	0.850	0.895	0.997	0.998

As seen in the external evaluation results in Table 4, performance metrics for the rule-based extractor were similar or better than those for the deep learning and machine learning models at both external testing sites. While VTExt’s precision and specificity scored high, sensitivity showed room for improvement (0.61 and 0.66 at PSH and University of Kentucky, respectively).

Error analysis of external testing results showed many deep learning model false negatives falling into 2 categories. Some errors can be attributed to overrepresentation of negated instances of certain VTE symptoms in the training dataset. This then makes the model more inclined to mark note sentences containing said symptoms as negative, even when the symptom is not negated. For the second category, less common terms used to describe relevant symptoms appear in testing data, for example, “malleoli” used in describing swelling of ankle. If such terms are not present in the training data, the model has no way of knowing they are relevant.

The rule-based model also produced false negatives, many belonging to one of two types. First, some errors can be attributed to double negation, which VTExt is not currently able to handle. For example, “SOB not resolved”—here, we see a VTE symptom, shortness of breath (SOB), followed by negating term “resolved.” However, “resolved” itself has been negated, and so this represents a positive instance. The second error type pertains to synonymous terms of phrases of VTE symptoms that are not currently included in the lexicon, for example, “black and blue area” as another way to phrase bruising. Since the phrase “black and blue area” is not part of the symptom lexicon, the rule-based model did not detect the symptom.

The results for the eCQM have been reported elsewhere [53]. The calculated rate of delayed VTE diagnosis was over 70% at both MGB and University of Kentucky, suggesting a clinically and practically meaningful measure for understanding delayed diagnosis rates across diverse health care sites.

**Table 4.** Performance of venous thromboembolism symptom extractor, deep learning, and machine learning models at University of Kentucky and Penn State Health sites.

Metric (95% CI)	PPV^a^	NPV^b^	Sensitivity	Specificity	Accuracy	AUROC^c^	AUPRC^d^
University of Kentucky							
VTExt^e^	1.00	0.75	0.66	1.00	0.83	—^f^	—
Deep learning	1.00 (1.00‐1.00)	0.63 (0.58‐0.68)	0.42 (0.35‐0.48)	1.00 (1.00‐1.00)	0.71 (0.67‐0.75)	0.71 (0.68‐0.74)	0.85 (0.83‐0.87)
XGBoost	0.98 (0.96‐1.00)	0.71 (0.66‐0.76)	0.60 (0.53‐0.66)	0.99 (0.97‐1.00)	0.79 (0.76‐0.83)	0.79 (0.76‐0.82)	0.89 (0.87‐0.91)
Logistic regression	0.95 (0.87‐1.00)	0.54 (0.49‐0.58)	0.16 (0.11‐0.20)	0.99 (0.98‐1.00)	0.57 (0.53‐0.62)	0.57 (0.55‐0.60)	0.76 (0.71‐0.80)
Random forest	1.00 (1.00‐1.00)	0.52 (0.48‐0.57)	0.08 (0.05‐0.12)	1.00 (1.00‐1.00)	0.54 (0.50‐0.58)	0.54 (0.52‐0.56)	0.77 (0.75‐0.79)
SVM^g^	0.98 (0.94‐1.00)	0.56 (0.51‐0.61)	0.22 (0.16‐0.27)	1.00 (0.99‐1.00)	0.61 (0.56‐0.65)	0.61 (0.58‐0.63)	0.79 (0.76‐0.82)
PSH							
VTExt	0.98	0.84	0.61	0.99	0.87	—	—
Deep learning	0.90 (0.85‐0.94)	0.82 (0.79‐0.84)	0.55 (0.49‐0.60)	0.97 (0.96‐0.98)	0.83 (0.81‐0.86)	0.76 (0.73‐0.79)	0.80 (0.77‐0.83)
XGBoost	0.87 (0.82‐0.91)	0.82 (0.80‐0.85)	0.58 (0.53‐0.63)	0.96 (0.94‐0.97)	0.83 (0.81‐0.86)	0.77 (0.74‐0.80)	0.79 (0.76‐0.83)
Logistic regression	0.86 (0.80‐0.91)	0.76 (0.73‐0.79)	0.37 (0.32‐0.43)	0.97 (0.96‐0.98)	0.77 (0.75‐0.8)	0.67 (0.65‐0.70)	0.72 (0.68‐0.76)
Random forest	0.95 (0.88‐1.00)	0.70 (0.67‐0.73)	0.12 (0.08‐0.15)	1.00 (0.99‐1.00)	0.71 (0.68‐0.74)	0.56 (0.54‐0.58)	0.68 (0.64‐0.71)
SVM	0.87 (0.82‐0.92)	0.77 (0.74‐0.80)	0.40 (0.35‐0.45)	0.97 (0.96‐0.98)	0.78 (0.76‐0.81)	0.69 (0.66‐0.71)	0.73 (0.70‐0.77)

a PPV: positive predictive value.

b NPV: negative predictive value.

c AUROC: area under the receiver operating characteristic curve.

d AUPRC: area under the precision-recall curve.

e VTExt: venous thromboembolism symptom extractor.

f Not available.

g SVM: support vector machine.

## Discussion

### Principal Findings

Much of the data not captured in structured EHR fields, like patient symptoms, are found in clinical notes [48]. In this study, we developed and validated a simple and generalizable NLP tool to identify and extract signs and symptoms of VTE from primary care notes through an iterative optimization process. VTExt is novel as the first NLP application linked to a nationally endorsed eCQM [65], helping to quantify the rate of delayed diagnosis of VTE in primary care. Through multiple rounds of optimization, VTExt showed robust performance and speed. Testing at two external sites demonstrated its ability to work well with different datasets and system configurations and its potential for optimizing quality measurement. We suggest that analysts familiar with their EHR and its local configurations could readily apply this NLP tool to their patient notes.

We learned several important lessons during optimization. Reducing the prevalence of false positives was crucial for improving extractor performance. In early rounds of validation, type B and type C errors often arose in long sentences due to a lack of constraint on the allowed search distance between a VTE symptom and a body part, or between a negating or contextual phrase and a symptom. We experimented with search distances of various lengths and found a distance of 150 characters struck a good balance of incorporating context without introducing too much noise, improving precision while maintaining high sensitivity.

We focused on primary care progress notes for developing and testing VTExt. Our external evaluation indicated that differences in note styles and hospital policies can affect performance. However, consistent performance observed between the 2 external sites highlighted VTExt’s strong generalizability. VTExt’s rule-based approach offers advantages including easier implementation, faster processing, and easier interpretation of results when compared with the tested machine learning and deep learning models. Error analysis also revealed further improvement opportunities for the symptom extractor. Working with collaborators at external sites to further refine VTExt to reduce false negatives would prove beneficial in improving sensitivity and NPV.

### Comparison With Previous Work

Shi et al [44] developed an NLP tool to detect postoperative VTE from free-text EHR notes. Internal validation demonstrated a sensitivity of 0.71 and specificity of 0.99. In the 2 health care systems tested, this NLP approach demonstrated superior performance in DVT surveillance than existing tools, and similar performance in PE surveillance compared with existing tools. Chapman et al [51] developed an NLP-based application to classify pulmonary angiography reports for document-level identification of PE, with test set performance resulting in sensitivity of 0.98 and PPV of 0.83. Sabra et al [66] incorporated Unified Medical Language System concept mapping into an NLP tool to generate feature vectors. These were then used to train and test an SVM machine learning model that achieved a PPV and sensitivity of 0.55 and 0.86, respectively. Work done by Jin et al [67] to identify VTE in inpatient notes using rule-based NLP methods highlights an approach that achieved similar performance to VTExt (0.90 sensitivity, 1.0 specificity), splitting notes into sentences, and then aggregating sentence-level information to make VTE inference at the sentence, document, and patient level. Although many of their tools would not be sufficient for our study’s goal of VTE symptom identification for quantifying delayed diagnosis, these studies show that NLP tools can effectively identify VTE events, and there is a need for more sensitive tools to identify VTE events using EHR progress notes in the primary care setting.

### Limitations

Our study has a number of limitations. First, VTExt is currently unable to handle misspellings in note text. Revising VTExt to handle misspellings would result in improved performance. Second, MGB was unable to view clinical note data used by external sites in the testing of VTExt in order to maintain patient data privacy. This reduced our ability to improve the tool’s generalizability, as MGB was unable to directly review output from the University of Kentucky and PSH other than performance metrics. Third, development and refinement of VTExt was based on 279 patient notes. While high performance was achieved, a wider dataset would provide additional context and understanding of the ways VTE symptoms are documented in clinical note text, allowing for further improvement of the tool.

### Future Directions

While a rule-based approach was simpler to implement, future improvements in accessible, high-performance LLMs could make them useful and feasible for quality measurement. These tools have already shown good results in extracting information from radiology reports [68], and could also be used to extract signs and symptoms from other types of clinical notes. Since LLMs are trained on large volumes of data, such an approach may generalize better across different health care systems and differently formatted notes when compared with a rule-based method. An LLM approach may more easily generalize to extracting symptoms from types of notes other than primary care progress notes, a logical future direction for research in this area. An immediate LLM-based approach was not pursued because we began this project in 2020 before there was mass public access to LLMs. While LLMs prove a promising direction for future work, the cost, time, and knowledge required to test such an approach at the collaborating sites were real limiting factors. In addition to an LLM approach, future work to improve model performance could include expanding the lexicon of symptom synonyms, as well as more robust handling of context and negation.

In addition to an LLM approach, future work to improve model performance could include expanding the lexicon of symptom synonyms, as well as more robust handling of context and negation.

### Conclusions

We developed a robust and efficient NLP-based tool, VTExt, to extract VTE-associated symptoms from primary care notes. VTExt achieved high sensitivity and specificity, performance that matches or exceeds that of deep learning models and demonstrates its reliability for clinical use. High sensitivity ensures that most patients with VTE symptoms are correctly identified, reducing the risk of missed or delayed diagnoses, which can have serious or fatal consequences. High specificity minimizes false positives, helping avoid unnecessary tests, anxiety, and resource use. Together, these metrics underscore VTExt’s clinical value in supporting timely, accurate identification of potential VTE cases from unstructured data.

VTExt’s generalizability across health care systems further supports its real-world applicability, enabling scalable deployment in diverse EHR environments. Its rule-based design facilitates transparency and ease of implementation, particularly for quality measurement initiatives such as tracking delayed diagnosis. Furthermore, the clinician-guided optimization process developed alongside VTExt provides a replicable framework for future NLP tool development and integration into clinical workflows, helping bridge the gap between EHR data and actionable insights for patient safety and care improvement.

## Supplementary material

10.2196/63720 Multimedia Appendix 1 Venous thromboembolism symptom lexicon.

10.2196/63720 Multimedia Appendix 2 Inclusion criteria *ICD-10* (*International Statistical Classification of Diseases, Tenth Revision*) and RxNorm codes.

10.2196/63720 Multimedia Appendix 3 Symptom prevalence, patient note level.

10.2196/63720 Multimedia Appendix 4 Deep learning and machine learning model parameters.

## Abbreviations

AUPRC: area under the precision-recall curve
AUROC: area under the receiver operating characteristic curve
BERT: bidirectional encoder representations from transformers
DVT: deep vein thrombosis
eCQM: electronic clinical quality measure
EDW: enterprise data warehouse
EHR: electronic health record
HIPAA: Health Insurance Portability and Accountability Act
*ICD-10*: *International Statistical Classification of Diseases, Tenth Revision*
LLM: large language model
MGB: Mass General Brigham
MIMIC: Medical Information Mart for Intensive Care
MTERMS: Medical Text Extraction, Reasoning and Mapping System
NLP: natural language processing
NPV: negative predictive value
PE: pulmonary embolism
PPV: positive predictive value
PSH: Penn State Health
SOB: shortness of breath
SVM: support vector machine
TF-IDF: term frequency—inverse document frequency
VTE: venous thromboembolism
VTExt: venous thromboembolism symptom extractor
