# Supplementary material for: An Extraction Tool for Venous Thromboembolism Symptom Identification in Primary Care Notes to Facilitate Electronic Clinical Quality Measure Reporting: Algorithm Development and Validation Study
Source: JMIR Med Inform. 2025 Aug 26;13:e63720. doi: 10.2196/63720 (PMC12387394; doi:10.2196/63720)
Supplement: Multimedia Appendix 1 [file medinform-v13-e63720-s001.docx]

**Appendix 1**. VTE symptom lexicon.

| **Location-Independent Symptoms** | **Location-Dependent Symptoms** | **Locations** |
| --- | --- | --- |
| cough | pain | chest* |
| hypotension | numbness | calf |
| lightheadedness | tingling | leg |
| shortness of breath | redness | foot |
| syncope | swelling |  |
| tachycardia | tenderness |  |
| hemoptysis | warmth |  |

*The “chest” location was only used when searching for the “pain” symptom.
