# Supplementary material for: An Extraction Tool for Venous Thromboembolism Symptom Identification in Primary Care Notes to Facilitate Electronic Clinical Quality Measure Reporting: Algorithm Development and Validation Study
Source: JMIR Med Inform. 2025 Aug 26;13:e63720. doi: 10.2196/63720 (PMC12387394; doi:10.2196/63720)
Supplement: Multimedia Appendix 2 [file medinform-v13-e63720-s002.docx]

**Appendix 2**. Inclusion criteria ICD 10 and RxNorm codes.

| **Concept (ICD 10)** | **ICD 10 Code** |
| --- | --- |
| acute kidney failure | N17.x |
| nephrotic syndrome | N04.x |
| chronic kidney disease | N18.x |
| proteinuria | R80.x |
| abnormality of albumin | R77.0 |
| other hypertrophic cardiomyopathy | I42.2 |
| chronic ischemic heart disease | I25.x |
| other cerebrovascular diseases | I67.x |
| nontraumatic intracerebral hemorrhage | I61.x |
| cerebral infarction | I63.x |
| embolism and thrombosis of unspecified artery | I74.9 |
| acute myocardial infarction | I21.x |
| acute cerebrovascular insufficiency | I67.81 |
| tachycardia, unspecified | R00.0 |
| heart failure | I50.x |
| left ventricular failure, unspecified | I50.1 |
| right (isolated) (ventricular) | I50.810 |
| atrial fibrillation and flutter | I48.x |
| pulmonary embolism | I26.x |
| deep vein thrombosis | I82.4x |

| **Imaging Procedure (CPT)** | **CPT Code** |
| --- | --- |
| Computed tomography, thorax, diagnostic; without contrast material | 71250 |
| Computed tomography, thorax, diagnostic; with contrast material(s) | 71260 |
| Computed tomography, thorax, diagnostic; without contrast material, followed by contrast material(s) and further sections | 71270 |
| Computed tomographic angiography, chest (noncoronary), with contrast material(s), including noncontrast images, if performed, and image postprocessing | 71275 |
| Computed tomographic angiography, abdomen, with contrast material(s), including noncontrast images, if performed, and image postprocessing | 74175 |
| Computed tomography, abdomen and pelvis; without contrast material | 74176 |
| Computed tomography, abdomen and pelvis; with contrast material(s) | 74177 |
| Computed tomography, abdomen and pelvis; without contrast material in one or both body regions, followed by contrast material(s) and further sections in one or both body regions | 74178 |
| Duplex scan of extremity veins including responses to compression and other maneuvers; complete bilateral study | 93970 |
| Duplex scan of extremity veins including responses to compression and other maneuvers; unilateral or limited study | 93971 |

| **Medication Name** | **RxNorm Medication ID** |
| --- | --- |
| HEPARIN (PORCINE) 1,000 UNIT/500 ML (2 UNIT/ML) IN 0.9 % NACL IV SOLN | 195216 |
| HEPARIN (PORCINE) 5,000 UNIT/1,000 ML IN 0.9 % SODIUM CHLORIDE IV SOLN | 127935 |
| HEPARIN (PORCINE) 2,000 UNIT/1,000 ML(2 UNIT/ML) IN 0.9 % NACL IV SOLN | 195061 |
| HEPARIN (PORCINE) 25,000 UNIT/250 ML IN 0.45 % SODIUM CHLORIDE IV SOLN | 15849 |
| HEPARIN (PORCINE) 2,500 UNIT/500 ML (5 UNIT/ML) IN 0.9 % NACL IV SOLN | 127235 |
| APIXABAN 5 MG (74 TABS) TABLETS IN A DOSE PACK | 197054 |
| APIXABAN 2.5 MG TABLET | 160879 |
| RIVAROXABAN 15 MG TABLET | 112834 |
| EDOXABAN 60 MG TABLET | 181388 |
| HEPARIN (PORCINE) 25,000 UNIT/250 ML (100 UNIT/ML) IN DEXTROSE 5 % IV | 15846 |
| WARFARIN 6 MG TABLET | 19434 |
| DABIGATRAN ETEXILATE 110 MG CAPSULE | 186948 |
| WARFARIN 5 MG INTRAVENOUS SOLUTION | 14959 |
| WARFARIN 4 MG TABLET | 21372 |
| ENOXAPARIN 150 MG/ML SUBCUTANEOUS SYRINGE | 31921 |
| APIXABAN 5 MG TABLET | 160996 |
| XARELTO 10 MG TABLET | 110251 |
| COUMADIN 5 MG TABLET | 1966 |
| WARFARIN 10 MG TABLET | 8748 |
| RIVAROXABAN 20 MG TABLET | 112835 |
| JANTOVEN 2 MG TABLET | 37042 |
| WARFARIN 1 MG TABLET | 11664 |
| EDOXABAN 15 MG TABLET | 181386 |
| XARELTO DVT-PE TREATMENT 30-DAY STARTER 15 MG(42)-20 MG(9) TABLET PACK | 179670 |
| WARFARIN 2.5 MG TABLET | 8750 |
| RIVAROXABAN 15 MG (42)-20 MG (9) TABLETS IN A STARTER PACK | 179663 |
| WARFARIN 7.5 MG TABLET | 8752 |
| WARFARIN 2 MG TABLET | 8749 |
| ELIQUIS 5 MG TABLET | 160997 |
| RIVAROXABAN 2.5 MG TABLET | 199549 |
| DABIGATRAN ETEXILATE 150 MG CAPSULE | 106491 |
| DABIGATRAN ETEXILATE 75 MG CAPSULE | 106490 |
| WARFARIN 3 MG TABLET | 19433 |
| RIVAROXABAN 10 MG TABLET | 110250 |
| ELIQUIS DVT-PE TREATMENT 30-DAY STARTER 5 MG (74 TABLETS) IN DOSE PACK | 197057 |
| PRASUGREL 10 MG TABLET | 98373 |
| ELIQUIS 2.5 MG TABLET | 160885 |
| EDOXABAN 30 MG TABLET | 181387 |
| WARFARIN 5 MG TABLET | 8751 |
| XARELTO 20 MG TABLET | 112837 |
| XARELTO 15 MG TABLET | 112836 |
| DALTEPARIN (PORCINE) 7,500 ANTI-XA UNIT/0.3 ML SUBCUTANEOUS SYRINGE | 108033 |
| ENOXAPARIN 100 MG/ML SUBCUTANEOUS SYRINGE | 105903 |
| FONDAPARINUX 10 MG/0.8 ML SUBCUTANEOUS SOLUTION SYRINGE | 108029 |
| DALTEPARIN (PORCINE) 18,000 ANTI-XA UNIT/0.72 ML SUBCUTANEOUS SYRINGE | 108036 |
| FONDAPARINUX 7.5 MG/0.6 ML SUBCUTANEOUS SOLUTION SYRINGE | 108028 |
| ENOXAPARIN 300 MG/3 ML SUBCUTANEOUS SOLUTION | 105940 |
| ENOXAPARIN 80 MG/0.8 ML SUBCUTANEOUS SYRINGE | 105902 |
| FONDAPARINUX 5 MG/0.4 ML SUBCUTANEOUS SOLUTION SYRINGE | 108027 |
| DALTEPARIN (PORCINE) 12,500 ANTI-XA UNIT/0.5 ML SUBCUTANEOUS SYRINGE | 108034 |
