# Supplementary material for: An Extraction Tool for Venous Thromboembolism Symptom Identification in Primary Care Notes to Facilitate Electronic Clinical Quality Measure Reporting: Algorithm Development and Validation Study
Source: JMIR Med Inform. 2025 Aug 26;13:e63720. doi: 10.2196/63720 (PMC12387394; doi:10.2196/63720)
Supplement: Multimedia Appendix 3 [file medinform-v13-e63720-s003.docx]

**Appendix 3**. Symptom prevalence, patient note level.

| **Symptom** | **Case Cohort** (279 Notes) | **Control Cohort** (50 Notes) |
| --- | --- | --- |
| cough | 47 | 4 |
| hypotension | 0 | 0 |
| lightheadedness | 0 | 0 |
| shortness of breath | 86 | 6 |
| syncope | 0 | 0 |
| tachycardia | 17 | 0 |
| hemoptysis | 0 | 0 |
| chest pain | 41 | 4 |
| calf pain | 15 | 0 |
| leg pain | 54 | 7 |
| foot pain | 11 | 1 |
| calf numbness | 0 | 0 |
| leg numbness | 1 | 0 |
| foot numbness | 0 | 0 |
| calf tingling | 0 | 0 |
| leg tingling | 2 | 0 |
| foot tingling | 0 | 0 |
| calf redness | 9 | 0 |
| leg redness | 4 | 0 |
| foot redness | 1 | 0 |
| calf swelling | 15 | 0 |
| leg swelling | 56 | 5 |
| foot swelling | 6 | 0 |
| calf tenderness | 10 | 0 |
| leg tenderness | 15 | 0 |
| foot tenderness | 0 | 0 |
| calf warmth | 0 | 0 |
| leg warmth | 2 | 0 |
| foot warmth | 0 | 0 |
