# Supplementary material for: An Extraction Tool for Venous Thromboembolism Symptom Identification in Primary Care Notes to Facilitate Electronic Clinical Quality Measure Reporting: Algorithm Development and Validation Study
Source: JMIR Med Inform. 2025 Aug 26;13:e63720. doi: 10.2196/63720 (PMC12387394; doi:10.2196/63720)
Supplement: Multimedia Appendix 4 [file medinform-v13-e63720-s004.docx]

**Appendix 4**. Deep learning and machine learning model parameters.

Deep Learning (Bio+Clinical BERT sequence classification model)

| **Parameter** | **Value** |
| --- | --- |
| Batch size | 10 |
| Learning rate | 5e-5 |
| Weight decay | 0.01 |
| Epochs (iterations) | 3 |

Logistic Regression

| **Parameter** | **Value** |
| --- | --- |
| Max iterations | 1000 |
| Learning rate | Optimal |
| Alpha | 1e-5 |

SVM

| **Parameter** | **Value** |
| --- | --- |
| Max iterations | 5000 |
| Learning rate | Optimal |
| Alpha | 1e-4 |

Random Forest

| **Parameter** | **Value** |
| --- | --- |
| N estimators | 500 |
| Max depth | None |

XGBoost

| **Parameter** | **Value** |
| --- | --- |
| Eta (learning rate) | 0.3 |
| Max depth | 100 |
| Gamma (min split loss) | 0.1 |
